# Twists and turns: A case report of cecal volvulus

**DOI:** 10.1002/ccr3.7936

**Published:** 2023-09-19

**Authors:** Aashish Sapkota, Aashna Batajoo, Samit Lamichhane, Alok Shrestha, Nidhi Bhatt

**Affiliations:** ^1^ Chitwan Medical College Chitwan Nepal

**Keywords:** cecal volvulus, gastro‐surgery, intestinal obstruction, whirl sign

## Abstract

We present a case of a 22‐year‐old male presenting in the emergency room with colicky abdominal pain, vomiting, and abdominal distension for which an early computed tomography scan was done and diagnosed as cecal volvulus. Following diagnosis case was managed promptly by laparotomy with right hemicolectomy and primary anastomosis.

## Graphical abstract

Gangrenous cecum, ascending colon and terminal ilium.

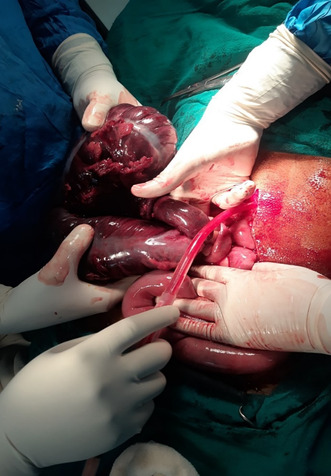

## 1 INTRODUCTION

Axial twisting of the cecum, ascending colon, and terminal ileum around their mesenteric pedicle is referred to as cecal volvulus.^1^ Cecal volvulus occurs in 1%–1.5% of all intestinal obstructions. The prognosis of the disease is poor with a mortality rate of 10%–40% depending on the bowel viability or gangrene.^2^ The prognosis of the disease depends on bowel viability, the patient's age, comorbid condition, and the time elapsed following the onset of symptoms, diagnosis, and intervention.^3^ Acute cecal volvulus can proceed to cecal gangrene and subsequent perforation, which can cause acute peritonitis if the diagnosis is delayed. Mostly diagnosis is delayed to its infrequent occurrence and nonspecific signs and symptoms. The two most important radiological methods for diagnosing a volvulus of the cecum are an abdominal X‐ray and an abdominal CT scan.^4^

We report a case of 22 year‐old male patient who presented to the emergency department with a chief complaint of periumbilical colicky abdominal pain diagnosed as cecal volvulus on abdominal CT scan. The case report is in line with SCARE^5^ guidelines.

## 2 CASE REPORT

A 22‐year‐old male patient nonsmoker, nonalcohol consumer, with no past comorbidities presented to the emergency department of our tertiary care center with the complaint of acute onset colicky abdominal pain for 19 h, around the periumbilical region which later became diffuse. Associated with vomiting three episodes nonbilious, non‐projectile, no passage of stool and flatus for 2 days, and gradual distention of abdomen. No history of fever, weight loss, trauma, and rectal bleeding. There was no past surgical history or history of any bowel pathologies in his family.

On examination, the patient was ill‐looking with stable vitals. The abdomen was moderately distended, tympanic upon percussion, and mild tenderness was noted on the right iliac region and hypogastrium without rigidity or rebound tenderness. Bowel sound was absent. On per rectal examination, the anal tone was normal, fingertip was stained with fecal particles.

He was referred to our center from the local hospital where he was managed symptomatically. Ultrasonography (USG) at the local hospital showed invagination of a bowel loop in another bowel loop in the right iliac fossa region forming hypoechoic concentric rings (target sign), likely intussusception.

**FIGURE 1:**
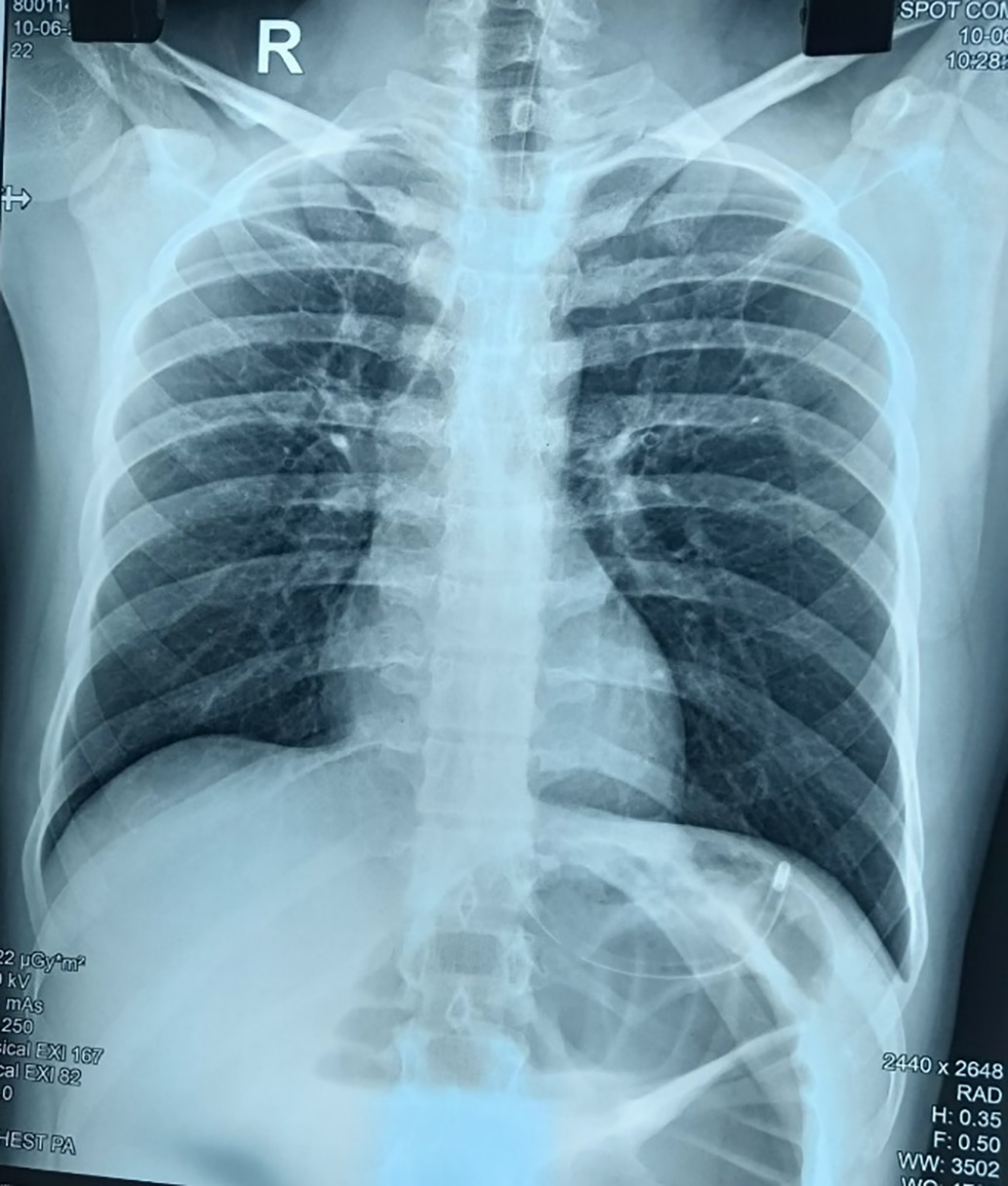
Plain X‐ray showing dilated colon reaching up to left hypochondrium.

**FIGURE 2:**
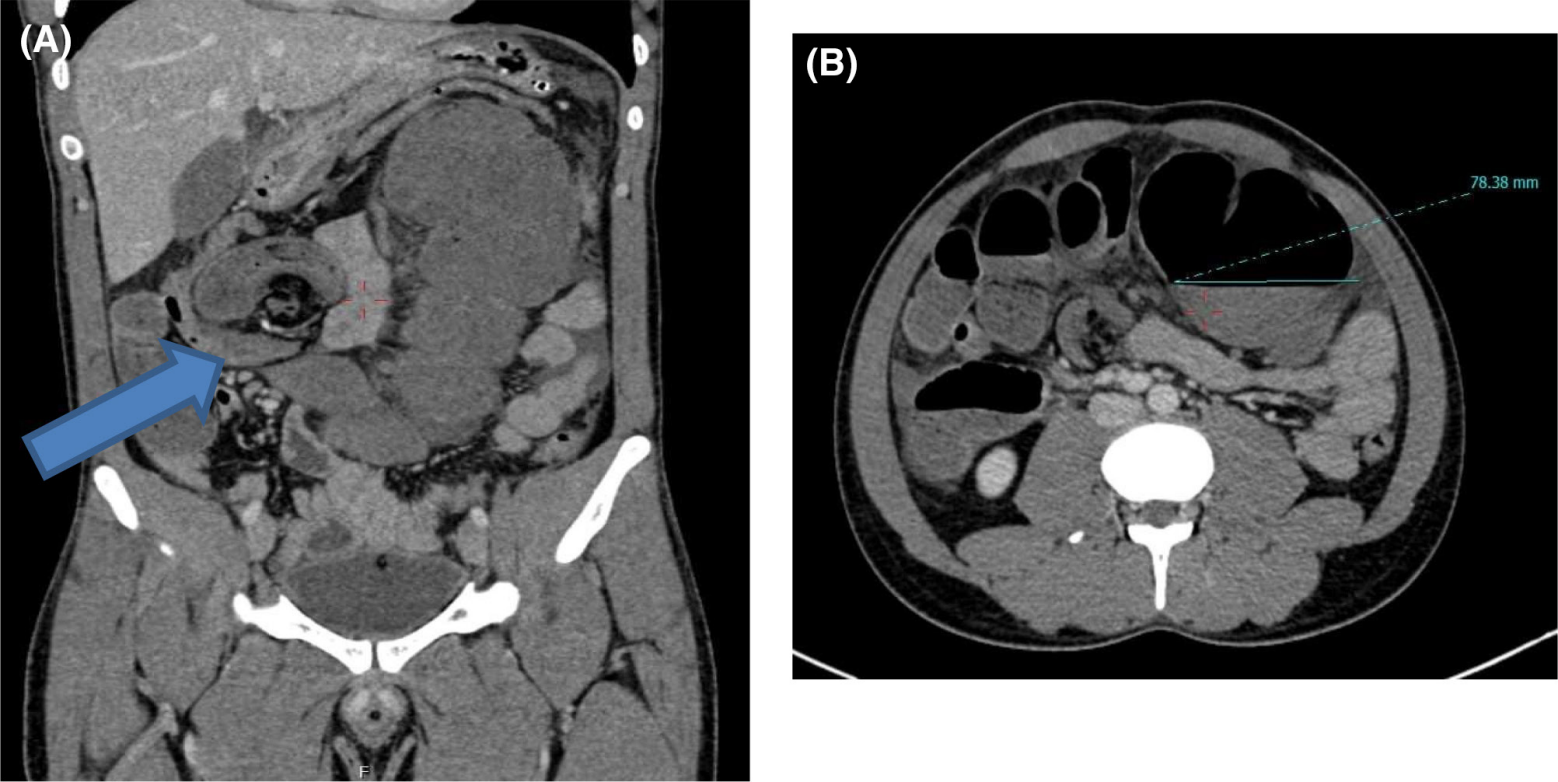
ct scan of abdomen and pelvis with contrast, (A) sagittal view showing dilated colon and whirlpool sign (arrow) (B) axial view showing dilated caecum.

On further investigation in our center, a plain film of the abdomen showed significant distention of the bowel with haustral folds reaching up to the left hypochondrium as in (Figure 1). Computed tomography (CT) abdomen pelvis suggested features likely of cecal volvulus with a high possibility for strangulation which is shown in (Figure 2A,B). Baseline investigations were within normal limits.

**FIGURE 3:**
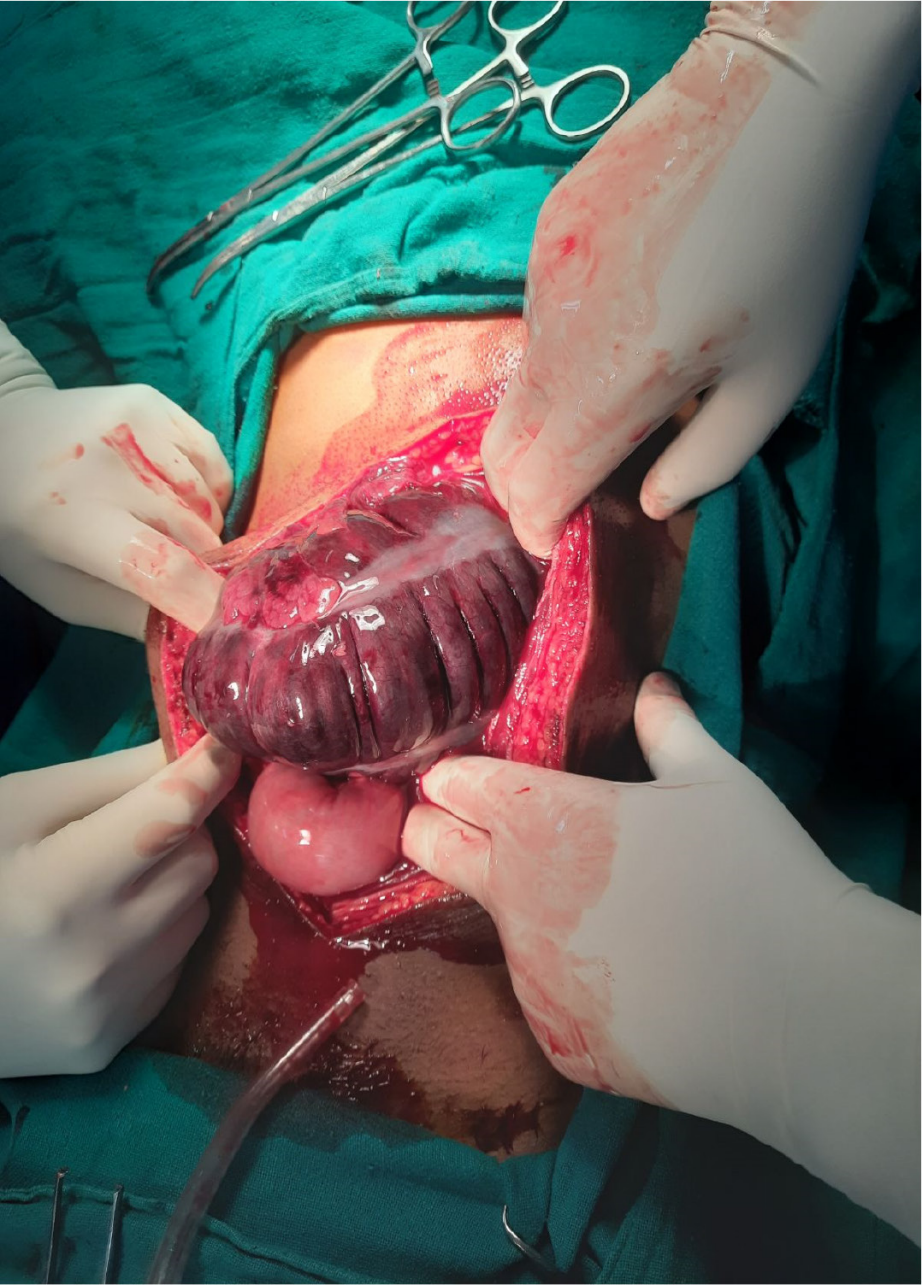
Intraoperative pictures showing: Gangrenous caecum.

**FIGURE 4:**
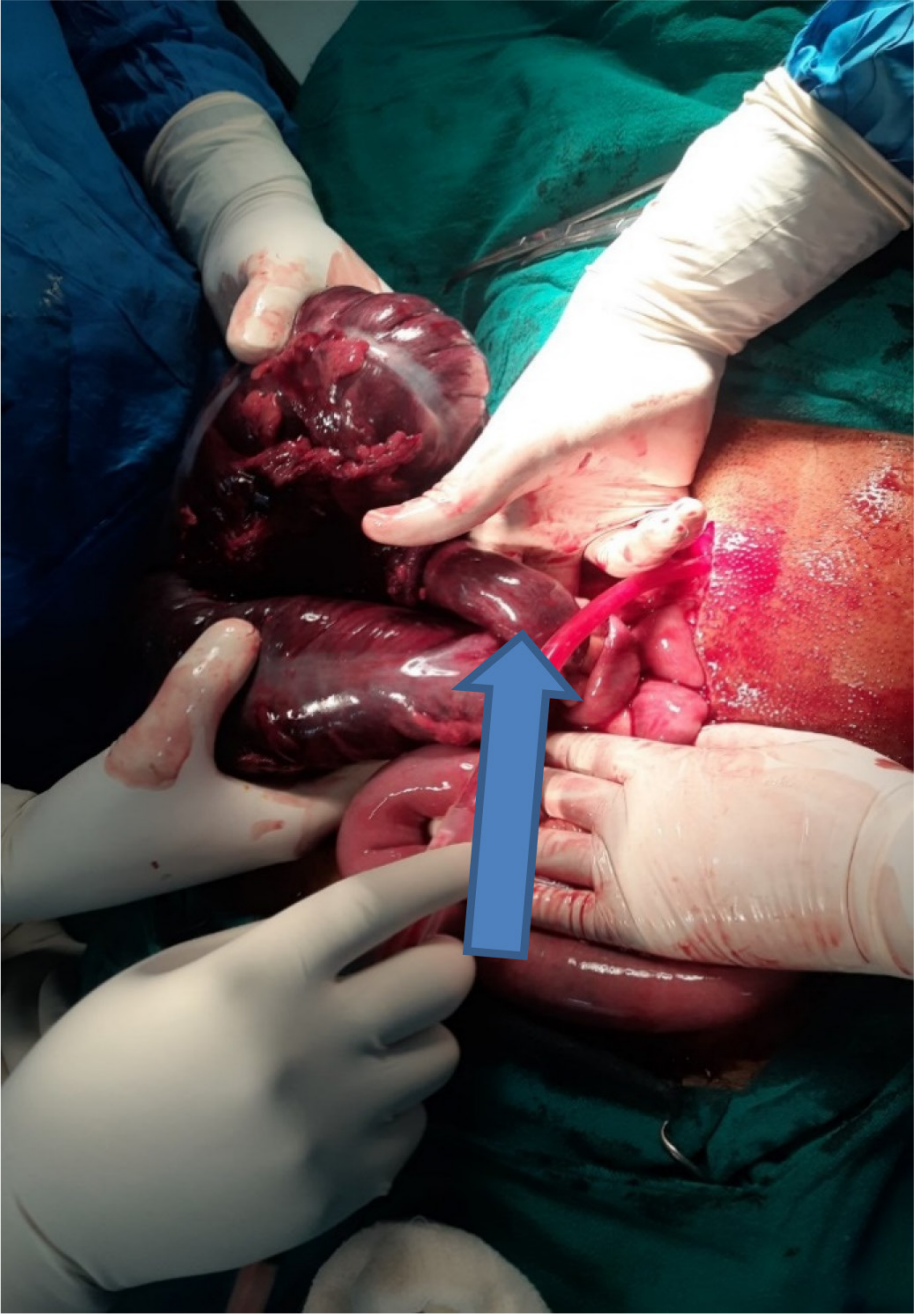
Gangrenous cecal volvulus (arrow: bowel loop twisting around the mesentery causing the gangrene), Gangrenous ascending colon and terminal ilium.

**FIGURE 5:**
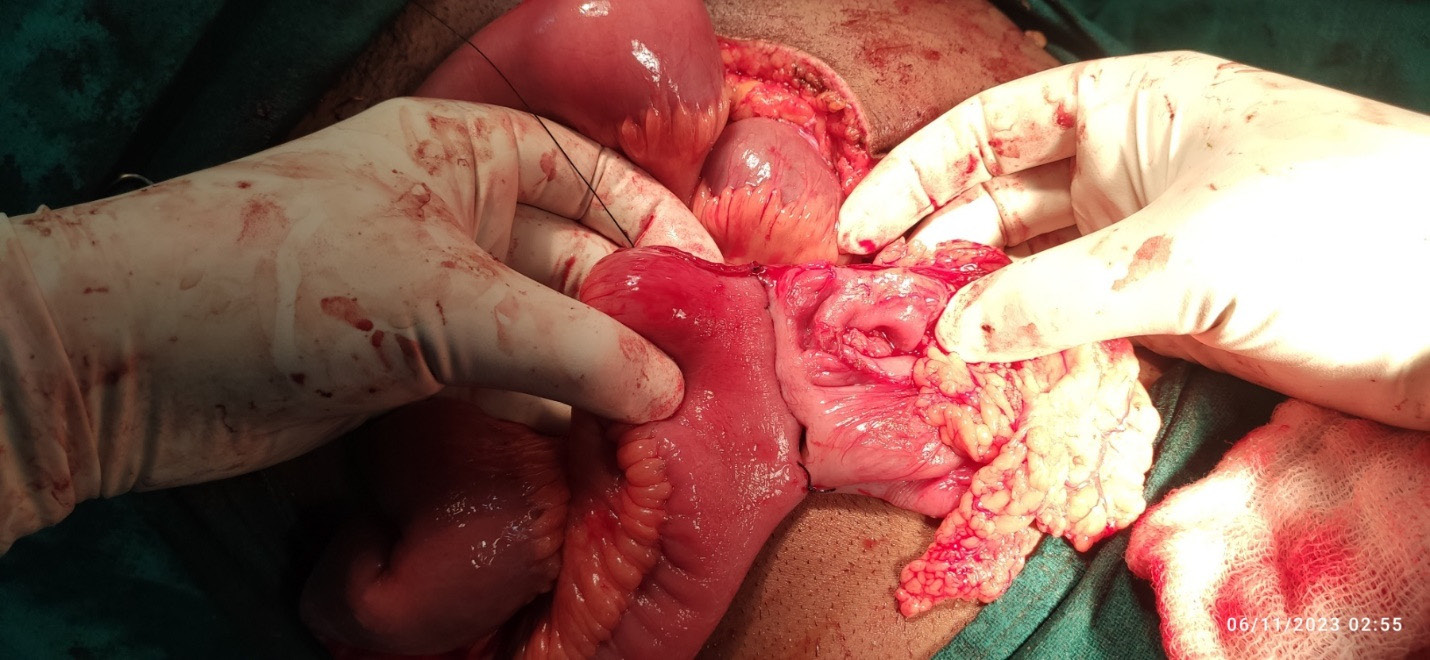
Side to side ileocolic anastomosis with linear stapler 60 and 100 cm followed by strengthening of the suture line with silk 3/0.

At the emergency room patient was managed conservatively with the administration of intraveno fluids, intravenous antibiotics, and pain was managed with intravenous analgesics following which the patient was shifted to the operation theater. The abdomen was opened in layers through a midline incision. Intraoperative, cecal volvulus was noted with gangrenous terminal ileum up to 10 cm plus gangrenous caecum extending up to mid‐transverse colon with ascetic fluid as shown in (Figures 3 and 4). Resection of the gangrenous part was done, and after that enterostomy was done in the ileum and along the tinea in the transverse colon, following which side‐to‐side ileocolic anastomosis was done with linear stapler 60 and 100 cm followed by strengthening of suture line with silk 3/0 and pelvic drain placement which is shown in (Figure 5). Remaining right colon was not attached with lateral abdominal wall.

The patient had two spikes of fever on second and third postoperative days. The postoperative period was complicated by wound dehiscence. Antibiotic cefuroxime was started along with dressing with Gentamycin and Metronidazole twice a day. On the 11th postoperative day secondary suturing under general anesthesia was done (Clavien‐Dindo Classification 3b). Postoperative recovery was uneventful hence, the patient was discharged on the 15th day. On follow‐up visits to the outpatient department, he was recovering well, with a complete return to normal daily activities. The histopathological report showed features consistent with volvulus. Granuloma formation, features of dysplasia, and malignancy were not seen in the sections examined.

## 3 DISCUSSION

In this casereport we presented a case of 22‐year‐old male presented with cecal volvulus managed by right hemicolectomy.

Cecal volvulus is one of the rare causes of intestinal obstruction accounting for 1%–1.5% of the cases and 25%–40% of all colonic volvulus.^6^ Although Nepal is considered to be present in volvulus belt, there are no significant studies showing the prevalence of cecal volvulus. The evidence of cecal volvulus is limited to few case reports and studies. A retrospective study on volvulus from mid‐western Nepal showed total 64 cases in duration of 5 years which included 86% of the cases as sigmoid volvulus and only 9% of the cases were of cecal volvulus.^7^, ^8^, ^9^ The cause of cecal volvulus is likely multifactorial. It has been widely associated with chronic constipation, abdominal masses, late‐term pregnancy, previous abdominal surgery, prolonged immobility, high fiber intake, paralytic ileus, and colonoscopy. Mobile cecum due to hypo‐fixation during the development of gut is supposed to be a cause in 11% of the cases of colonic volvulus as suggested by cadaveric examination.^6^, ^10^

Cecal volvulus has variable presentation ranging from recurrent abdominal pain, acute abdominal pain, abdominal distension, absolute constipation, vomiting, and shock. Recurrent abdominal pain is seen in cases of mobile cecum syndrome, which can progress to acute cecal volvulus as well as can resolve spontaneously.^6^, ^11^, ^12^, ^13^, ^14^ Acute cecal volvulus presents with acute abdominal pain with a clinical picture similar to acute intestinal obstruction rapidly progressing to strangulation and possible perforation leading to hemodynamic instability and shock.^15^, ^16^

Laboratory investigations are not specific for the diagnosis of cecal volvulus. Imaging modalities are the mainstay of diagnosis. Cecal volvulus is characterized radiographically on X‐ray by a dilated gas‐filled viscus that is typically found ectopically in the left upper quadrant or mid‐abdomen. Caecum retains haustral markings on X‐ray film in comparison to the sigmoid volvulus.^17^ Abdominal CT scan is the best modality for the diagnosis of cecal volvulus with high sensitivity and specificity. “Whirl sign” on a CT scan is an important sign for the diagnosis of cecal volvulus which contains a spiraled loop of collapsed caecum and engorged mesenteric vessels whereas similar “whirl sign” can also be observed in sigmoid volvulus where whirl sign is caused by dilated sigmoid colon around its mesocolon and vessels. Along with the whirl sign other features seen on the CT scan are the central appendix sign, abnormal cecal position, severe cecal distension, and mesenteric engorgement in a case of cecal volvulus.^18^, ^19^, ^20^

Management strategies for cecal volvulus include surgical resection, endoscopic reduction, and cecopexy. Endoscopic reduction carries a significant risk of recurrence whereas cecopexy is only suitable for volvulus with viable viscus. Hence these methods of management are highly individualized on a case‐to‐case basis.^6^, ^21^, ^22^, ^23^, ^24^, ^25^ American College of Colon and Rectum Surgeons recommends segmental resection of nonviable or gangrenous cecum for the management of cecal volvulus.^26^ Resection can be done via both open as well as laparoscopic approaches. However laparoscopic approaches are rarely done.^27^ The main advantage of resection is it reduces the chance of recurrence and decreases mortality.^14^ Resection with ileostomy and distal mucous fistula formation is advocated for cases with large gangrenous area and cecal perforation whereas primary anastomosis following resection is reserved for cases with patchy gangrenous caecum.^6^ Resection of the gangrenous part of the colon along with colopexy of the right colon remnant had been advocated to prevent recurrent volvulus.^28^

The timely diagnosis and management of the cecal volvulus is essential as the condition has significant mortality if not managed early.^29^ Patient with untreated acute cecal volvulus develops intestinal strangulation and perforation ultimately leading to peritonitis and shock.^6^ Thus, for preventing serious complications of cecal volvulus they must be diagnosed early and managed promptly.

## 4 CONCLUSION

Cecal volvulus is an acute life‐threatening condition with multifactorial causation including anatomical abnormalities, previous surgeries, lifestyle‐associated factors like prolonged immobility, and high fiber diet intake. CT scan is the most useful method of diagnosis as it confirms the diagnosis allowing prompt operative procedure for definitive management and reduction of mortality of the cases presenting in the emergency room.

## AUTHOR CONTRIBUTIONS

**Aashish Sapkota:** Conceptualization; writing – original draft. **Aashna Batajoo:** Data curation; supervision; visualization. **Samit Lamichhane:** Conceptualization; supervision; writing – original draft. **Alok Shrestha:** Conceptualization; supervision; writing – review and editing. **Nidhi Bhatt:** Data curation; visualization; writing – review and editing.

## CONFLICT OF INTEREST STATEMENT

The authors have no conflict of interest to declare.

## CONSENT

Written informed consent was obtained from the patient for the publication of this case report and accompanying images. A copy of the written consent is available for review on request.

## Data Availability

All the clinical records and consent are available for review on request.
